# DNAJB13 polymorphisms and association with idiopathic asthenozoospermia in Sichuan, China

**DOI:** 10.1186/s12610-025-00295-w

**Published:** 2025-12-18

**Authors:** Jiaoyu He, Zhuo Zhang, Yishan Ding, Chunlan Cheng, Ning Li, Tianjun Li, Xianqiong Zhao, Chengyue Wang, Xianping Ding

**Affiliations:** 1 Renji Medical Research Center, West China School of Medicine, Sichuan University, Sichuan University affiliated Chengdu Second People’s Hospital, Chengdu Second People’s Hospital, Chengdu, Sichuan 610021 P.R. China; 2 Key Laboratory of Bio-Resources and Eco-Environment of Ministry of Education, College of Life Sciences, Sichuan University, Chengdu, Sichuan 610065 P.R. China; 3 Chongqing Nanchuan Biotechnology Research Institute, Bio-Resource Research and Utilization Joint Key Laboratory of Sichuan and Chongqing, Chongqing 408400 Nanchuan, P.R. China; 4 Department of Medical Genetics and Prenatal Diagnosis, Sichuan Provincial Women’s and Children’s Hospital/The Affiliated Women’s and Children’s Hospital of Chengdu Medical College, 610000, Sichuan 610000 Chengdu, P. R. China; 5 Department of Laboratory Medicine, Sichuan Provincial Maternity and Child Health Care Hospital, Sichuan 610000 Chengdu, P.R. China; 6 Senior High School Department, Mapleleaf International School- Chongqing, Chongqing 402100 Yongchuan, P.R. China; 7 Dazhou Vocational and Technical College, Sichuan 635000 Dazhou, P.R. China

**Keywords:** DNAJB13 gene, Idiopathic asthenozoospermia, Sperm axonemal protein, Single nucleotide polymorphisms, Bioinformatics analysis, Gène DNAJB13, Asthénozoospermie idiopathique, Protéine axonémale du Spermatozoïde, Polymorphismes mononucléotidiques, Analyse bio-informatique

## Abstract

**Background:**

The axonemal co-chaperone gene DNAJB13 is essential for sperm motility and structural integrity. This study aimed to investigate the association between coding variants in DNAJB13 and idiopathic asthenozoospermia (IAZS) in a cohort from Sichuan, China.

**Methods:**

Sanger sequencing of all DNAJB13 exons was performed in 130 patients with IAZS and 120 fertile controls with strictly normal semen parameters according to World Health Organization (WHO) 5th edition criteria. Detected variants were analyzed for genotype and allele frequencies. The potential impact of a novel missense variant was assessed using evolutionary conservation analysis across mammalian orthologs and in silico prediction tools for structural and splicing effects.

**Results:**

Six coding variants were identified. Three variants (c.T279C, c.C882T, c.G927A) were common single nucleotide polymorphisms (SNPs ) with no significant differences between cases and controls. A novel missense variant, c.T272G (p.V91G), was detected exclusively in patients. Genotype distribution and allele frequency of this variant differed significantly between groups. Valine 91 was highly conserved, and computational modeling predicted that the p.V91G substitution would destabilize protein structure, disrupt hydrophobic core architecture, potentially interfere with RNA splicing regulatory elements, and impair DNAJB13 co-chaperone function in axonemal assembly.

**Conclusions:**

The c.T272G (p.V91G) in DNAJB13 is significantly associated with IAZS in the studied cohort and represents a potential high-risk genetic factor. Predicted detrimental effects on protein structure and function suggest a novel genetic mechanism underlying impaired sperm motility. Functional validation and replication studies in diverse populations are necessary to confirm its pathogenic role.

## Résumé

**Contexte:**

Le gène axonémal co-chaperonne DNAJB13 est essentiel à la mobilité et à l’intégrité structurelle du spermatozoïde. Cette étude visait à étudier l’association entre les variants codants de DNAJB13 et l’asthénozoospermie idiopathique (AZSI) dans une cohorte du Sichuan, en Chine. Le séquençage de Sanger de tous les exons de DNAJB13 a été réalisé chez 130 patients avec AZSI et 120 témoins fertiles avec des paramètres de sperme strictement normaux selon les critères de 5e édition de l’OMS. Les variants détectés ont été analysés pour les fréquences génotypiques et alléliques. L’impact potentiel d’une nouvelle mutation faux-sens du variant a été évalué à l’aide d’analyses de conservation évolutive sur les orthologues de mammifères, et d’outils de prédiction in silico pour les effets structurels et d’épissage.

**Résultats:**

Six variants codants ont été identifiés. Trois variants (c.T279C, c.C882T, c.G927A) étaient des polymorphismes nucléotidiques simples courants (SNPs) sans différences significatives entre les cas et les témoins. Un nouveau variant faux-sens, c.T272G (p.V91G), a été détecté exclusivement chez les cas. La distribution du génotype et la fréquence de l’allèle de ce variant différaient significativement entre les groupes. La Valine 91 était très conservée ; la modélisation informatique prédisait que la substitution de p.V91G déstabiliserait la structure des protéines, perturberait l’architecture du noyau hydrophobe, pourrait potentiellement interférer avec les éléments régulateurs de l'épissage de l’ARN, et altérerait la fonction de co-chaperonne de DNAJB13 dans l'assemblage axonémal.

**Conclusions:**

c.T272G (p.V91G) chez DNAJB13 est significativement associé à l’AZSI dans la cohorte étudiée, et représente un facteur génétique potentiellement à haut risque. Les effets néfastes prédits sur la structure et la fonction des protéines suggèrent un nouveau mécanisme génétique sous-jacent à la motilité altérée des spermatozoïdes. Des études de validation fonctionnelle et de réplication dans diverses populations sont nécessaires pour confirmer son rôle pathogène.

## Introduction

 Human infertility is a multifactorial disorder influenced by environmental, behavioral, and genetic factors, affecting 10–25% of reproductive-aged couples in the Asian region. Male factors are implicated in approximately 40–50% of these cases [1, 2]. Male infertility is characterized by impaired semen parameters, including azoospermia (absence of sperm in the ejaculate), oligozoospermia (sperm concentration < 15 × 10⁶/mL), asthenozoospermia (AZS, progressive motility < 32%), and teratozoospermia (normal morphology < 4%) [3]. IAZS, characterized by a reduction in sperm motility without an identifiable cause, represents a significant diagnostic challenge in male reproductive medicine. While oligozoospermia is the most common etiological category of male infertility, IAZS accounts for 20–30% of all cases. Notably, over 50% of these IAZS cases lack a definitive pathogenic explanation despite comprehensive diagnostic evaluation [4, 5]. Although substantial efforts have been made to identify genes associated with male infertility resulting from sperm abnormalities, the underlying mechanisms remain largely unknown. This highlights a critical need for further research, particularly into the genetic basis of IAZS [2].

A mature spermatozoon comprises two principal components: the head, housing the genetic material, and the tail (flagellum), which is responsible for its motility during fertilization [6, 7]. Consequently, structural defects in any component, particularly within the axoneme of the flagellum, can impair sperm function and lead to infertility. The proper formation and function of a spermatozoon depend on the coordinated expression of more than 200 proteins. Among these, members of the heat shock protein 40 (HSP40) family play critical roles in maintaining axonemal integrity [7–9]. DNAJB13, also known as testis spermatogenesis apoptosis-related protein 6 (TSARG6), is a type II HSP40/DnaJ protein. It localizes to the radial spokes (RSs) of the axoneme, where it is essential for maintaining sperm motility. Furthermore, DNAJB13 mediates the formation of the annulus—a critical junction between the midpiece and principal piece of the flagellum—through dynamic interactions with septin 4 (SEPT4) during spermiogenesis [10, 11].

The canonical “9 + 2” axoneme consists of nine peripheral microtubule doublets that encircle a central pair of microtubules sheathed by the central apparatus (CA). Motility is orchestrated through coordinated interactions between dynein arms and RSs [12–14]. This structure enables flagellar beating via coordinated interactions between inner dynein arms (IDAs), outer dynein arms (ODAs), and RSs. The specific composition of these components contributes to the structural diversity of axonemes across different types of mammalian motile cilia. Composed of T-shaped hexameric complexes anchored by stalks to the central apparatus, RSs act as mechanochemical sensors. They convert mechanical stimuli into biochemical signals to regulate dynein-driven microtubule sliding [15]. Notably, DNAJB13 is the final RS component to be incorporated into the axoneme during spermiogenesis and is crucial for maintaining the structural integrity of the central apparatus in both motile cilia and flagella [10].

RSs are composed of at least 23 proteins, and mutations in these components result in a subtype of Primary Ciliary Dyskinesia (PCD) characterized by central pair (CC) defects [13, 16, 17]. Dysfunction of DNAJB13 compromises axonemal assembly by destabilizing the interaction between RSs and the CC, consequently leading to flagellar abnormalities and infertility [14, 18]. The protein’s high expression in ciliated tissues—such as the testis, trachea, lung, and oviduct—combined with its exclusive localization to mature sperm flagella, highlights its specialized role in sperm motility. Collectively, these attributes establish DNAJB13 as a pivotal candidate gene for elucidating the pathogenesis of IAZS, particularly in light of its dual function in both RS assembly and CC maintenance.

Despite the established role of DNAJB13 in axonemal integrity and sperm motility, its specific contribution to IAZS remains poorly understood. The molecular mechanisms underlying its involvement in IAZS pathogenesis are particularly elusive, especially its interactions with other axonemal components such as the RSs and the CC. To address this knowledge gap, we conducted a study to systematically analyze DNAJB13 polymorphisms in a cohort of 130 IAZS patients from Sichuan, China. To ensure the specificity of our analysis, we excluded individuals with other known genetic causes of infertility, including Y-chromosome microdeletions and mutations in *DNAH* [9, 19, 20], *SEPT4* [18] and *MTHFR* [21]. This research aims to provide a robust theoretical foundation for elucidating the pathogenic role of DNAJB13 in IAZS and to inform the development of future therapeutic strategies.

## Patients and methods

### Patients and controls sample resources

A total of 130 IAZS patients (31.0 ± 4.8 years; range 25–40 years) and 120 fertile male controls (32.1 ± 4.8 years; 25–40 years) were prospectively recruited from the Affiliated Hospital of Sichuan Genital Hygiene Research Center (Sichuan, China) between March 2015 and May 2017. Patients with cryptorchidism, varicocele, epididymitis, testicular tumors, genital trauma, prostatitis, obstruction of the vas deferens, endocrine hypogonadism, karyotype anomalies and Y chromosome microdeletions or environmental exposures (tobacco, alcohol, radiation, chemicals)—were excluded. Controls were defined as men with normal semen parameters per WHO 5th edition criteria, proven paternity through natural conception, and no history of infertility-related disorders.

### Semen and karyotype analysis

Semen samples were collected via masturbation after 3–5 days of abstinence, with sperm parameters (concentration, total motility, viability, and round cell count) assessed per WHO 5th edition guidelines. Inclusion criteria required sperm concentration ≥ 15 × 10⁶/mL, total progressive motility (A + B grades) ≥ 40%, and normal morphology ≥ 4% in fresh ejaculates, with a minimum of three independent analyses confirming these parameters [3]. Karyotyping was performed using standard G-banding cytogenetics, analyzing ≥ 20 metaphase spreads for routine cases and ≥ 50 spreads for suspected chromosomal abnormalities to ensure diagnostic accuracy.

### Genomic DNA extraction

Genomic DNA was extracted from human spermatozoa using the Easy-Pure Blood Genomic DNA Kit (TransGen, Beijing, China) following optimized protocols. Sperm pellets were resuspended in sterile water and lysed with 500 µL binding buffer containing 20 µL proteinase K (10 mg/mL) and 10 µL RNase A (10 mg/mL), followed by 10-minute incubation at room temperature. The lysate was then applied to a centrifugal column for DNA binding, with subsequent washing steps using 500 µL pre-mixed binding/wash buffer (containing 70% ethanol) to eliminate contaminants. Purified DNA was eluted in 100 µL preheated (60 °C) elution buffer (EB) and quantified via NanoDrop spectrophotometry (A260/A280 ≥ 1.8). All samples were stored at −20 °C until downstream analysis.

### The analysis of Y microdeletions detection

Multiplex PCR was performed to detect AZF microdeletions in 130 IAZS patients using six sequence-tagged sites (STSs) spanning the Yq11 AZF regions: *AZFa* (*sY86*, *sY84*), *AZFb* (*sY127*, *sY134*), *AZFc* (*sY254*, *sY255*), with *SRY* (*sY14*) as an internal control. Two multiplex reactions were designed: Multiplex 1 amplified *sY84*, *sY134*, and *sY255*; Multiplex 2 targeted *SRY*, *sY86*, *sY127*, and *sY254*. Reactions included normal male (positive control), female (negative control), and nuclease-free water (no-template control). Amplified products underwent agarose gel electrophoresis for size-based analysis, with samples showing expected band patterns progressing to downstream analysis.

### PCR amplification and genotyping

The complete coding sequence and flanking intronic regions of the *DNAJB13* were amplified via PCR using an A200 Gradient Thermal Cycler (Long Gene) with primers designed by Primer 5 (sequences listed in Table 1). The 25 µL reaction system contained 1× Taq buffer, 1.5 mM Mg²⁺, 0.25 mM dNTPs, 0.1 µM forward/reverse primers, 2 U Taq DNA polymerase (Takara), and 200 ng genomic DNA. Amplification conditions were: 95 °C for 5 min (initial denaturation), followed by 35 cycles of 94 °C for 1 min (denaturation), gradient annealing (54 °C/61°C/63°C for 1 min, respectively), 72 °C for 1 min (extension), and a final 72 °C extension for 5 min. PCR products were resolved on 2% agarose gels (Abgent Biotechnology) with ethidium bromide staining (120 V, 35 min) for size verification. Bidirectional Sanger sequencing was performed using the ABI 3730XL DNA Analyzer (Applied Biosystems) with BigDye Terminator v3.1 Cycle Sequencing Kit (Sangon Biotech), followed by sequence alignment and mutation analysis via DNAMAN software (Lynnon BioSoft).

**Table 1:** Primer sequences of 8 exons in the *DNAJB13* gene

Exon	Primer	Primer sequence	Size (bp)	PCR product (bp)
1	DNAJB13-1 F	5’GTCAGGAGCAACCAAGGAAG3’	20	279
	DNAJB13-1R	5’CAAGAAGTGAAAGGAGGAACAA3’	22	
2	DNAJB13-2 F	5’AGGCAGGAGTGAACAGAGTG3’	20	298
	DNAJB13-2R	5’GGATGCTTCCTCGTTATTGG3’	20	
3	DNAJB13-3 F	5’AGCCCCTCCACCTATCTCAG3’	20	302
	DNAJB13-3R	5’AAAACTACTCAACGAAAACATC3’	22	
4	DNAJB13-4 F	5’TAGGATGCTGGGTGTT3’	16	376
	DNAJB13-4R	5’GCAGGAGATAGGTGGC3’	16	
5	DNAJB13-5 F	5’ATCCCATAGAGCATCTTTCACG3’	22	302
	DNAJB13-5R	5’TCCTCCTCAGTCTTCCCACC3’	20	
6	DNAJB13-6 F	5’TGGAGACAAGTAGAGGCAGGAA3’	22	262
	DNAJB13-6R	5’ATGGCAGGTGGTTGTGGGAG3’	20	
7	DNAJB13-7 F	5’CCTCCTCAGCCCCACCTTTG3’	20	308
	DNAJB13-7R	5’TCCTTTTGCCAGGTGATTGC3’	20	
8	DNAJB13-8 F	5’CCTCCTCAGCCCCACCTTTG3’	20	413
	DNAJB13-8R	5’TCCTTTTGCCAGGTGATTGC3’	20	

a F means forward primer; b R means reverse primer; c The primers of DNAJB13 gene were designed by PRIMER version 5.0 based on the DNAJB13 reference sequences (GenBank number: FR751039) and synthesized by Sangon Biotech (Shanghai, China)

### Physical and chemical properties prediction and statistical analysis

The evolutionary conservation of DNAJB13 was analyzed across six species (Homo sapiens, Pan troglodytes, Macaca fascicularis, Rattus norvegicus, Aotus nancymaae) using WebLogo (http://weblogo.berkeley.edu/) [22] with sequences retrieved from NCBI. Missense mutation pathogenicity was evaluated through four computational platforms: PolyPhen-2 (http://genetics.bwh.harvard.edu/pph2) [23], SIFT (http://sift.jcvi. org) [24], SNAP2 (http://www.rostlab.org/services/SNAP) [25] and PROVEAN (http://provean.jcvi.org) [26] Online software, which predict structural destabilization, evolutionary intolerance, and functional impact.

RNA splicing alterations were assessed by ASSP (http://wangcomputing.com/assp/) and RNAsnp (http://rth.dk/resources/rnasnp/) [27, 28], focusing on splice site disruption and exon skipping events. Structural analysis of wild-type and mutant DNAJB13 proteins was performed using Novopro (https://www.novopro.cn/tools/) and Protein Model Portal (http://www.proteinmodelportal.org/) [29], while ExPASy-ProtScale (https://web.expasy.org/protscale) [30] and I-Mutant 2.0 (http://folding.biofold.org/i-mutant/i-mutant 2.0.html) [31] evaluated hydrophobicity changes and thermal stability (ΔΔG >1 kcal/mol indicates destabilization).

Genotype frequencies and Hardy-Weinberg equilibrium (HWE) were compared between patients and controls using Pearson’s chi-squared test. Unconditional logistic regression quantified variant genotype-associated risks through odds ratios (ORs) and 95% confidence intervals (CIs). Statistical significance was defined as *P* < 0.05. All analyses were conducted using SPSS 20.0 [32].

## Result

### The semen parameters comparison analysis results

The clinical characteristics of IAZS patients and fertile controls are summarized in Table 2. Statistically significant differences were observed between groups for sperm concentration (38.0 ± 17.6 × 10⁶/mL vs. 62.8 ± 16.4 × 10⁶/mL, *P* = 0.018), rapid progressive motility (7.6 ± 6.2% vs. 37.6 ± 3.8%, *P* < 0.001), and total progressive motility (16.9 ± 9.8% vs. 75.5 ± 10.8%, *P* < 0.001), whereas age showed no intergroup disparity (31.2 ± 6.3 years vs. 34.9 ± 3.4 years, *P* = 0.487). All statistical analyses were performed using Welch’s t-test to account for unequal variances, with significance defined as *P* < 0.05.

**Table 2:** The age and semen parameters of asthenozoospermic group and controls

Sample parameters	Control group (*n* = 120)^a^	Asthenozoospermia group (*n* = 130)^a^	*P* ^b^
Age (year)	34.9 ± 3.4	31.2 ± 6.3	0.487
Sperm concentration (10^6^/mL)	62.8 ± 16.4	38.0 ± 17.6	0.018
Rapid progressive motility (%)	37.6 ± 3.8	7.6 ± 6.2	<0.001
Total progressive motility (%)	75.5 ± 10.8	16.9 ± 9.8	< 0.001

All analyses were conducted using SPSS 20.0

^a^means the data are presented as mean ± SD

^b^means the comparison between groups was done with the student’s t-test; *P* < 0.05 was considered statistically significant

### DNAJB13 sequence polymorphism analysis results

Genetic analysis of the DNAJB13 coding sequence in 130 patients with IAZS identified six non-synonymous nucleotide substitutions. These included c.C87T and c.T106C (exon 2), which are known common SNPs, and c.T279C (exon 3), c.C882T, and c.G927A (exon 8), which were classified as common population variants based on database frequencies. A novel missense variant, c.T272G (p.V91G), was identified exclusively in the patient cohort. Specifically, this variant was present in 5 of the 130 patients (3.85%, all heterozygous) but was absent in all healthy controls. This difference in allele frequency was statistically significant (*P* = 0.03). The complete genotype distribution is detailed in Table 3, showing significant differences between IAZS patients and controls. This c.T272G variant results in a valine-to-glycine substitution at amino acid position 91, a change that is predicted to disrupt the chaperone function of DNAJB13, which is critical for proper sperm flagellar assembly.1.002–1.037, *P* = 0.031). This missense mutation (c.T272G) induces an amino acid substitution at position 91 (Val→Gly), potentially disrupting DNAJB13’s chaperone function in sperm flagellar assembly.

**Table 3:** Genotype and allele frequencies of T272G in DNAJB13 among idiopathic asthenozoospermia and control

Genotype/Allele	Controls (120)	Cases (130)	Odds ratio (95%CI)	*P* value
272TT	(100%) 120	(96%) 125		
272TG	0	(4%) 5	1.040 (1.005–1.076)	0.030
272(GG)	0	(0%) 0		
272(TG + GG)	0	(4%) 5	1.040 (1.005–1.076)	0.030
272T	(100%) 120	(98.08%) 255		
272G	0	(1.92%) 5	1.020 (1.002–1.037)	0.031

All analyses were conducted using SPSS 20.0

*CI* means confidence interval; *P* < 0.05 was considered statistically significant

Genotype frequencies and Hardy-Weinberg equilibrium (HWE) were compared between patients and controls using Pearson’s chi-squared test, Unconditional logistic regression quantified variant genotype-associated risks through odds ratios (ORs) and 95% confidence intervals (CIs)

### DNAJB13 conservation and pathogenicity analysis

**Fig. 1:**
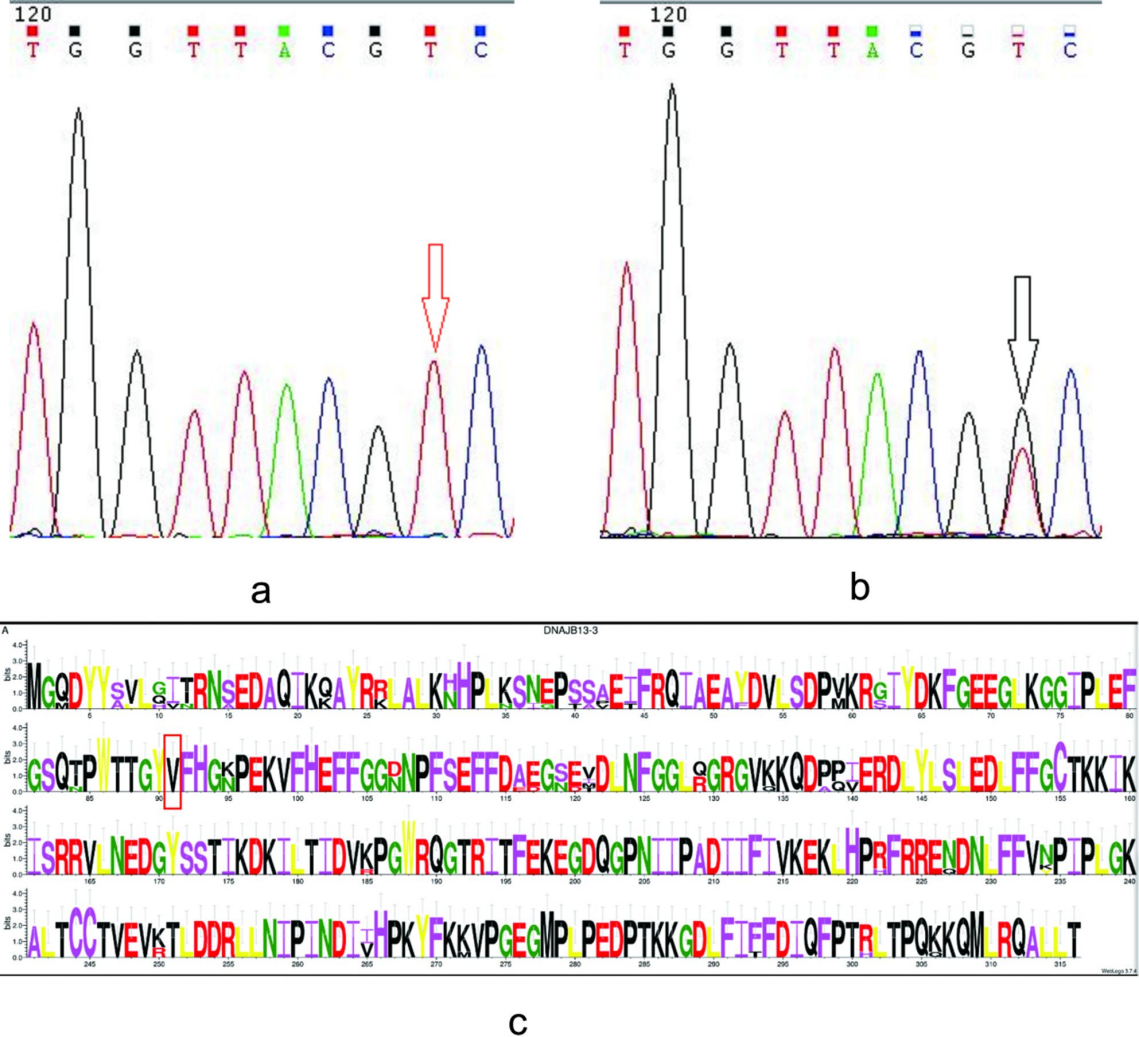
Mutation and conservation analysis of DNAJB13 Note: a means sequence analysis of the 272 T > G in the DNAJB13 with different allele expression in Wild-type homozygous genotype; b means sequence analysis of the 272 T > G in the DNAJB13 with different allele expression in Heterozygous genotype; c means sequence alignment of the DNAJB13 in the six different species, and the 91th amino-acid is framed with black box

Conservation analysis of DNAJB13 across six species revealed that position 91 resides within an evolutionarily conserved domain spanning invertebrates to higher vertebrates (Fig. 1). The valine residue at this position (91 V) demonstrated strict conservation, with no observed substitutions in reference databases. Computational pathogenicity predictions using PolyPhen-2, SIFT, SNAP2, and PROVEAN servers uniformly classified the c.T272G (p.V91G) missense mutation as likely pathogenic (Table 4).

**Table 4:** Pathogenicity prediction results of the DEFB126 variants

Nucleotide	Amino acid	PolyPhen-2	SIFT	SNAP	PROVEAN
C.T272G	P. V91G	possibly-damaging	Damaging	Effect	Deleterious

Nucleotide means Nucleotide mutation; Amino acid means Amino-acid substitution; Polyphen-2 prediction scores range from 0 to 1 with high scores indicating probably or possibly damaging; SIFT means Sorting Intolerant from Tolerant, scores vary between 0 and 1, and scores close or equal to 0 are predicted to be damaging; SNAP2 predict score ranges from − 100 strong neutral prediction to + 100 strong effect prediction that reflects the likelihood of this specific mutation to alter the native protein function; f PROVEAN scores lower than − 2.5 (cutoff) are predicted to be deleterious

### The c.T272G (V91G) effect on DNAJB13 RNA splicing and structure

**Fig. 2:**
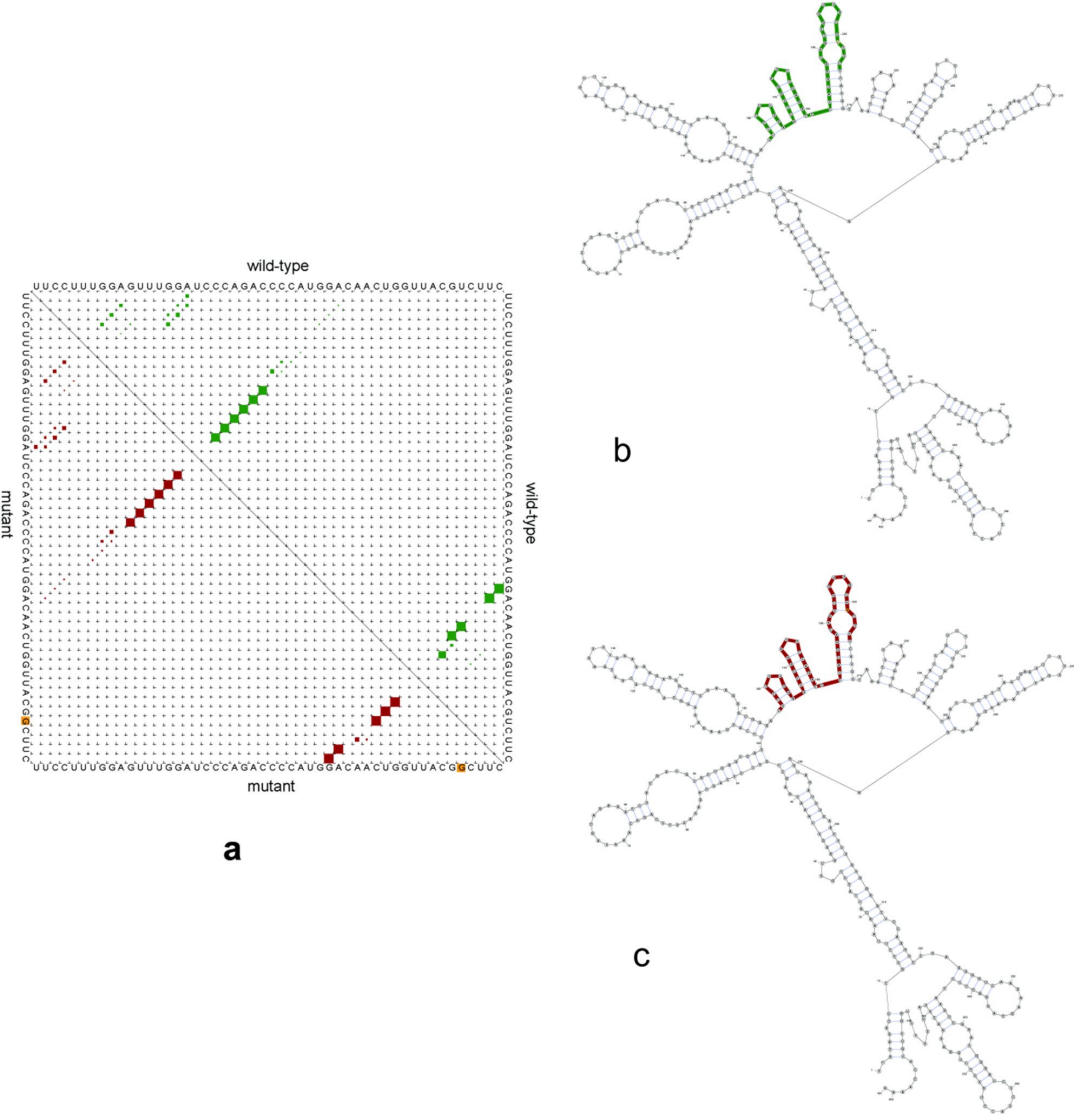
The effect of c. T272G on RNA structure Note: a shows the probability of wild-type and mutated sequences, polymorphic nucleotides are shown by yellow colour; b means the optimal secondary structure of the wild-type RNA sequence (156 to 205 nt highlighted), the minimum free energy of the wild type = −131.70 kcal/mol; c means the optimal secondary structure of the mutant RNA sequence (156 to 205 nt highlighted), the minimum free energy of the mutant RNA = −133.20 kcal/mol

Analysis using the Neural Network Splice Site Prediction Tool revealed that the c.T272G (p.V91G) mutation did not alter canonical splice site parameters (position, putative splice site, sequence composition, or scoring metrics) between wild-type and mutant transcripts. However, significant differences were observed in regulatory elements: Intron GC, Alt./Cryptic, Constitutive, Confidence (220 bp window), and Alt./Cryptic, Constitutive, Confidence (250 bp and 294 bp windows) (Tables 5, 6). Despite the absence of direct motif disruption in these regions, the mutation perturbs splicing regulatory elements. RNAsnp analysis (156–205 nt window) ‘revealed a subtle structural divergence (distance metric = 0.0698, P = 0.3976) between wild-type and mutant RNAs. Despite the statistical insignificance of the divergence, the T→G substitution significantly altered RNA stability by decreasing the minimum free energy (ΔG) from − 131.70 kcal/mol (wild-type) to −133.20 kcal/mol (mutant), corresponding to a ΔΔG of −1.5 kcal/mol. This energetic stabilization likely enhances base-pairing interactions within the stem-loop structure (Fig. 2), potentially impairing its dynamic flexibility required for DNAJB13’s chaperone activity during sperm flagellar assembly (Fig. 2).

**Table 5:** Effect of c. T272G on selective splicing sites (Reference)

Position (bp)	Putative splice site	Sequence	Score ^a^	Activations^b^			
				Intron GC^a^	Alt./Cryptic	Constitutive	Confidence^b^
125	Alt. isoform/cryptic acceptor	ccgtcttcagCAGAGATTTT	2.4	0.557	0.943	0.054	0.942
128	Alt. isoform/cryptic acceptor	tcttcagcagAGATTTTCAG	3.912	0.557	0.923	0.074	0.92
220	Alt. isoform/cryptic donor	GGCCTGAAGGgtgggattcc	9.404	0.529	0.766	0.172	0.776
250	Alt. isoform/cryptic acceptor	tggatcccagACCCCATGGA	2.699	0.529	0.6	0.384	0.361
294	Alt. isoform/cryptic donor	ACCTGAAAAGgtgttccacg	6.884	0.457	0.774	0.171	0.779
331	Alt. isoform/cryptic donor	AACCCCTTCAgtgagttttt	12.035	0.514	0.571	0.34	0.405
350	Alt. isoform/cryptic acceptor	tttgatgcagAAGGAAGTGA	6.287	0.457	0.841	0.157	0.813
353	Alt. isoform/cryptic acceptor	gatgcagaagGAAGTGAGGT	2.575	0.429	0.882	0.114	0.87
355	Alt. isoform/cryptic donor	GCAGAAGGAAgtgaggtaga	5.68	0.6	0.955	0.032	0.966
360	Alt. isoform/cryptic donor	AGGAAGTGAGgtagatttga	7.842	0.6	0.92	0.055	0.941
388	Alt. isoform/cryptic acceptor	ggggctccagGGCCGAGGGG	3.291	0.457	0.687	0.3	0.563
529	Alt. isoform/cryptic acceptor	caccatcaagGACAAGATCC	4.615	0.486	0.581	0.397	0.317
559	Alt. isoform/cryptic donor	GTGAAGCCCGgttggaggca	4.665	0.6	0.854	0.111	0.87
629	Alt. isoform/cryptic acceptor	atcccagcagACATCATTTT	2.882	0.614	0.812	0.18	0.778
649	Alt. isoform/cryptic acceptor	catcgtaaagGAGAAGCTAC	4.357	0.5	0.85	0.143	0.832
675	Constitutive acceptor	gcttccgcagGGAGAATGAC	9.569	0.529	0.228	0.758	0.699
744	Constitutive donor	CACTGTGGAGgtgaggaccc	11.483	0.486	0.329	0.59	0.443
816	Alt. isoform/cryptic donor	CTTCAAGAAGgtgccagggg	9.557	0.571	0.579	0.346	0.402
892	Alt. isoform/cryptic acceptor	cgacatccagTTCCCCACCC	6.795	0.557	0.973	0.026	0.973
916	Constitutive acceptor	cacaccccagAAGAAGCAGA	5.421	0.571	0.303	0.687	0.559

The effect of c.T272G on splice sites was predicted using Alternative Splice Site Predictor (ASSP)

^a^Scores reflect splice site strength: ^a^PSSM for acceptor sites and an MDD model for donor sites. Intron GC content was calculated for the adjacent 70 nt

^b^Activations are backpropagation network outputs for classification, where high values for one class and low for another indicate good classification. Confidence measures the difference between activations, ranging from 0 (undecided) to 1 (perfect)

**Table 6:** Effect of c. T272G on selective splicing sites (Mutant)

Position (bp)	Putative splice site	Sequence	Score^a^	Activations^b^			
				Intron GC^a^	Alt./Cryptic	Constitutive	Confidence^b^
125	Alt. isoform/cryptic acceptor	ccgtcttcagCAGAGATTTT	2.4	0.557	0.943	0.054	0.942
128	Alt. isoform/cryptic acceptor	tcttcagcagAGATTTTCAG	3.912	0.557	0.923	0.074	0.92
220	Alt. isoform/cryptic donor	GGCCTGAAGGgtgggattcc	9.404	0.543	0.739	0.193	0.739
250	Alt. isoform/cryptic acceptor	tggatcccagACCCCATGGA	2.699	0.529	0.596	0.39	0.346
294	Alt. isoform/cryptic donor	ACCTGAAAAGgtgttccacg	6.884	0.457	0.762	0.181	0.762
331	Alt. isoform/cryptic donor	AACCCCTTCAgtgagttttt	12.035	0.514	0.571	0.34	0.405
350	Alt. isoform/cryptic acceptor	tttgatgcagAAGGAAGTGA	6.287	0.457	0.841	0.157	0.813
353	Alt. isoform/cryptic acceptor	gatgcagaagGAAGTGAGGT	2.575	0.429	0.882	0.114	0.87
355	Alt. isoform/cryptic donor	GCAGAAGGAAgtgaggtaga	5.68	0.6	0.955	0.032	0.966
360	Alt. isoform/cryptic donor	AGGAAGTGAGgtagatttga	7.842	0.6	0.92	0.055	0.941
388	Alt. isoform/cryptic acceptor	ggggctccagGGCCGAGGGG	3.291	0.457	0.687	0.3	0.563
529	Alt. isoform/cryptic acceptor	caccatcaagGACAAGATCC	4.615	0.486	0.581	0.397	0.317
559	Alt. isoform/cryptic donor	GTGAAGCCCGgttggaggca	4.665	0.6	0.854	0.111	0.87
629	Alt. isoform/cryptic acceptor	atcccagcagACATCATTTT	2.882	0.614	0.812	0.18	0.778
649	Alt. isoform/cryptic acceptor	catcgtaaagGAGAAGCTAC	4.357	0.5	0.85	0.143	0.832
675	Constitutive acceptor	gcttccgcagGGAGAATGAC	9.569	0.529	0.228	0.758	0.699
744	Constitutive donor	CACTGTGGAGgtgaggaccc	11.483	0.486	0.329	0.59	0.443
816	Alt. isoform/cryptic donor	CTTCAAGAAGgtgccagggg	9.557	0.571	0.579	0.346	0.402
892	Alt. isoform/cryptic acceptor	cgacatccagTTCCCCACCC	6.795	0.557	0.973	0.026	0.973
916	Constitutive acceptor	cacaccccagAAGAAGCAGA	5.421	0.571	0.303	0.687	0.559

The effect of c.T272G on splice sites was predicted using Alternative Splice Site Predictor (ASSP)

^a^Scores reflect splice site strength: ^a^PSSM for acceptor sites and an MDD model for donor sites. Intron GC content was calculated for the adjacent 70 nt

^b^Activations are backpropagation network outputs for classification, where high values for one class and low for another indicate good classification. Confidence measures the difference between activations, ranging from 0 (undecided) to 1 (perfect)

### Impact of V91G (c.T272G) on protein structural integrity, stability, and hydrophilicity

**Fig. 3:**
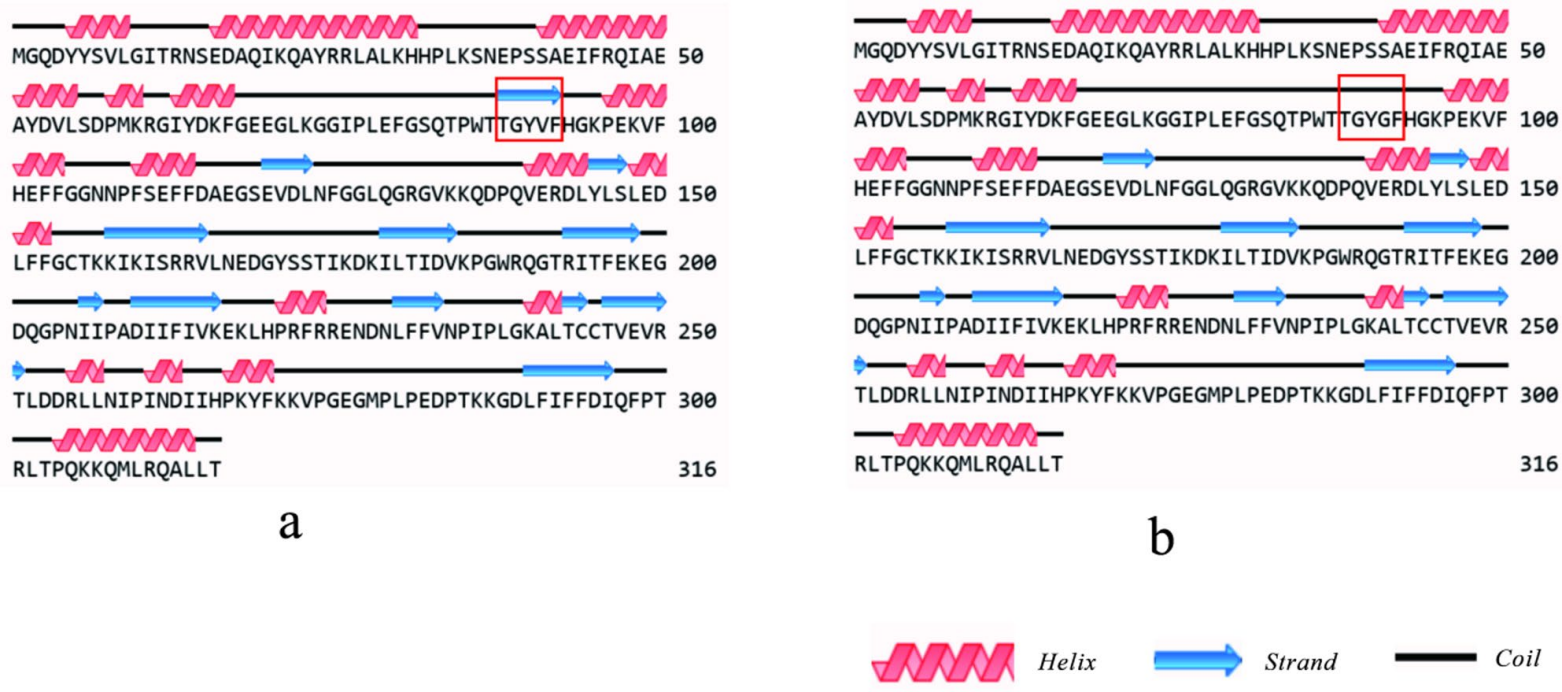
The effect of V91G on DNAJB13 protein secondary structure Note: a means the wild-type DNAJB13 protein, b means the mutant DEFB126 protein of V91G. The differences in DNAJB13 protein secondary structure are marked by red boxes

**Fig. 4:**
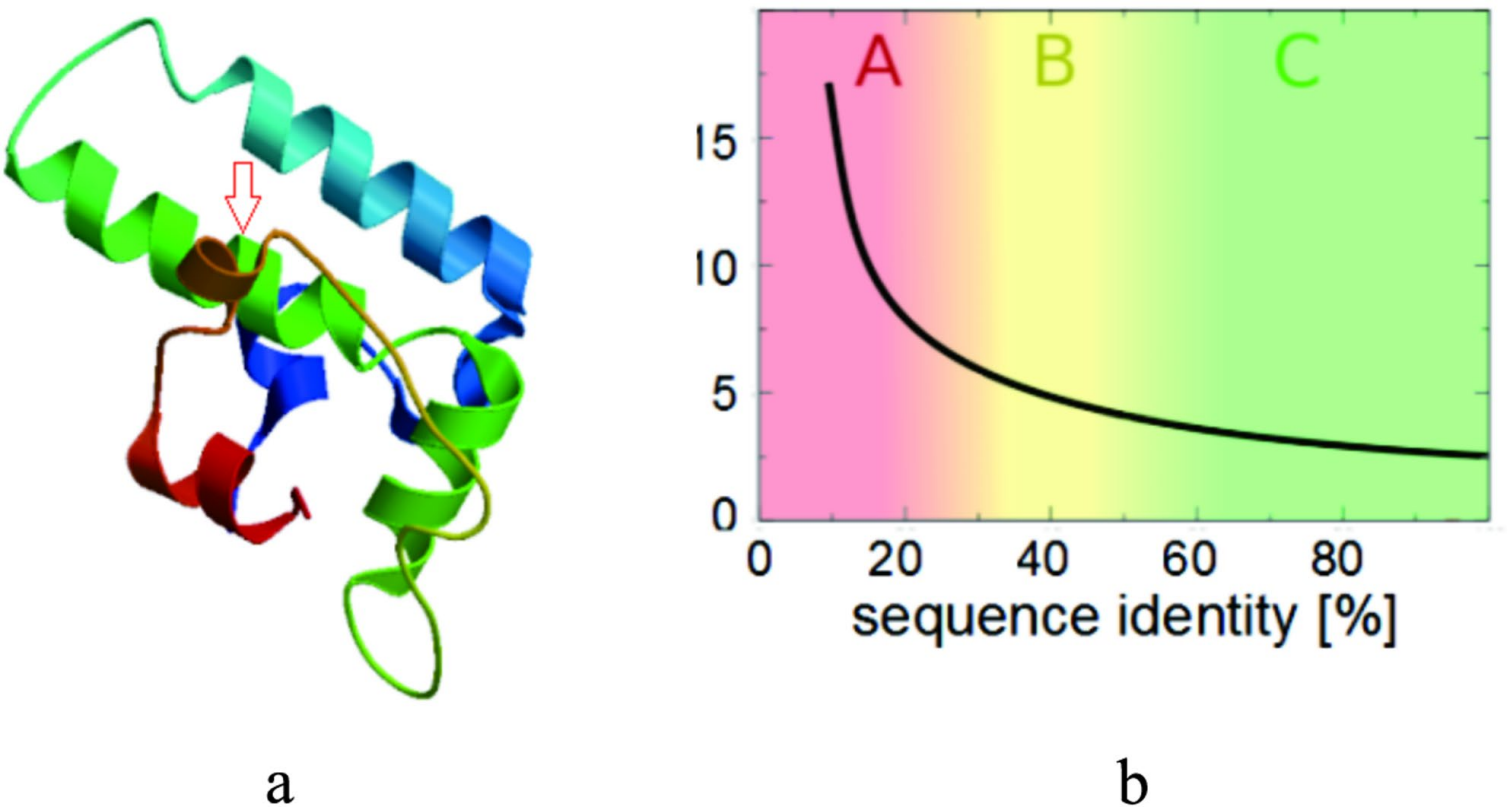
Protein structure model of DNAJB13 was given from the protein model portal Note: a means the location of mutation (V91G) in DNAJB13 protein 3D structure (V91G was marked by red arrow), b means this model is based on Target-template sequence alignment of 40% sequence identity

**Fig. 5:**
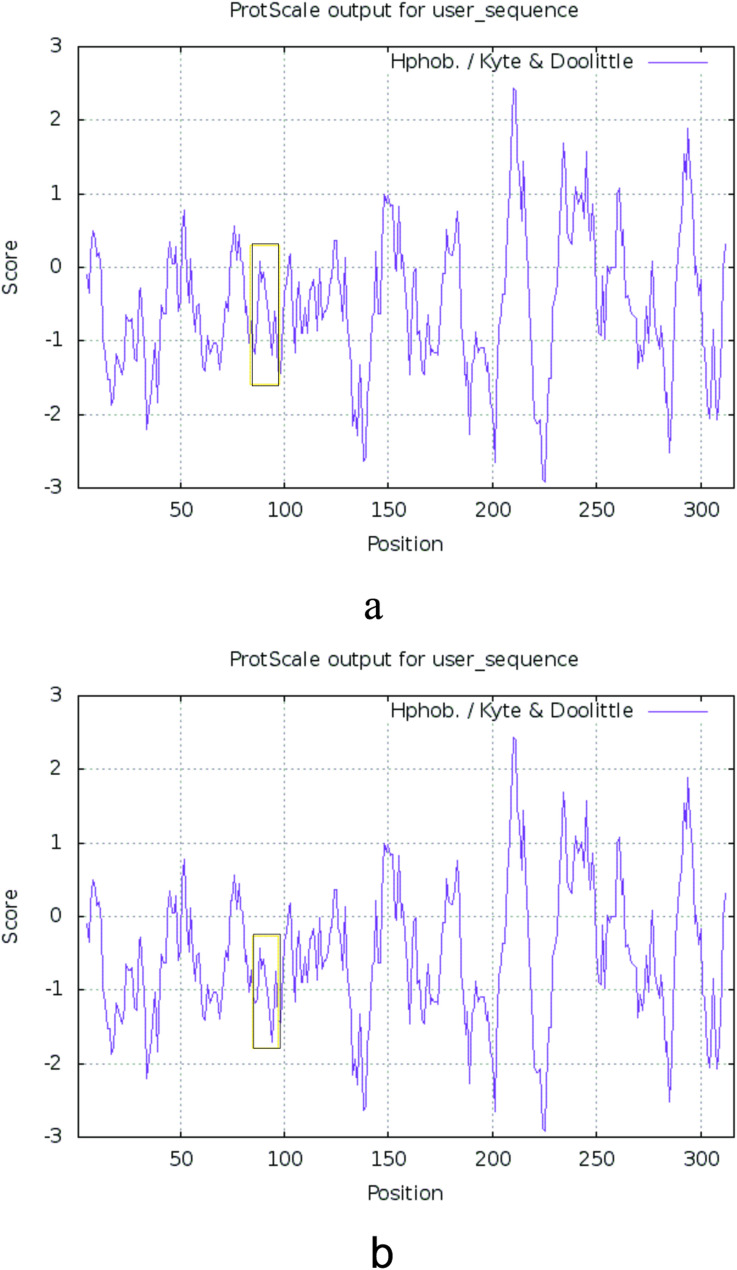
Hydrophobicity plot for DNAJB13 protein Note: a means the hydrophobicity of the wild-type DNAJB13 protein, b means the hydrophobicity of the DNAJB13 protein with V91G mutant. The areas of difference in hydrophobicity were shown in black boxes, and the hydrophobicity score of DNAJB13 V91G mutant was lower than the wild type

Structural analysis of DNAJB13 was performed using Novopro and Protein Model Portal, revealing that the c.T272G (p.V91G) mutation disrupts the β-strand spanning residues 88–92 (TGYVF) within the middle β-sheet domain (Fig. 3). This secondary structural alteration eliminates critical hydrogen bonds between V91 and adjacent residues (Fig. 3), leading to conformational strain in the tertiary fold (Fig. 4). Thermodynamic analysis by I-Mutant 3.0 indicates reduced structural stability in the mutant protein, with its DDG decreased to −4.50 kcal/mol (Table 7). ExPASy-ProtScale analysis detected altered hydrophobic residue distribution in the β-sheet region (Fig. 5), suggesting compromised structural integrity due to disrupted hydrophobic interactions.

**Table 7:** Effect of V91G on protein stability

Nucleotide	Amino acid	I-Mutant 2.0		
		Stability	RI	ΔΔG (kcal/mol)
c.T272G	p. V91G	Decrease	9	−4.50

Nucleotide means Nucleotide mutation, Amino-acid means Amino-acid substitution. DDG = DG (New Protein)-DG (Wild Type) in Kcal/mol, DDG < 0 means stability decrease, DDG > 0 menas stability increase

## Discussion

### DNAJB13: a dual-function regulator of sperm flagella

IAZS, is characterized by severely reduced sperm motility after excluding known causes like infections, varicocele, and common genetic defects [18]. Successful fertilization depends on sperm motility for traversing the female reproductive tract and penetrating the oocyte. While mutations in axonemal genes (e.g., DNAH1, RSPH, IFT) are known to impair motility by disrupting microtubule or mitochondrial function [7, 19, 33–36], the molecular basis of IAZS remains largely unclear.​.

DNAJB13 is an evolutionarily conserved protein located in the sperm flagellum’s fibrous sheath and mitochondrial sheath. It acts as a critical hub, coordinating two essential processes: (1) structural regulation, where it mediates interactions between axonemal components to ensure proper flagellar bending [14, 18]; and (2) bioenergetic regulation, where it optimizes mitochondrial ATP production [37]. Therefore, disruption of DNAJB13 can cause both structural disorganization and metabolic failure, leading to impaired sperm motility and male infertility. The importance of this gene is further highlighted in Primary Ciliary Dyskinesia (PCD), an autosomal recessive ciliopathy, where DNAJB13 variants account for 12–18% of cases and cause similar defects in sperm tail structure and energy metabolism [10, 38].

### Identification of a rare DNAJB13 variant (c.T272G, p.V91G) in IAZS patients

In this study, we identified a significant association between IAZS and a rare missense variant in DNAJB13: c.T272G, which results in a p.V91G substitution. Notably, this variant was present in 5 of the 130 IAZS patients (3.85%) but was completely absent in the 120 fertile controls, a difference that is statistically significant (*P* = 0.03; OR = 1.040, 95% CI = 1.005–1.076). This finding is biologically plausible, as DNAJB13 is a highly conserved HSP40 co-chaperone essential for sperm flagellar assembly and motility [39, 40]. The p.V91G substitution replaces a bulky valine with glycine, the smallest amino acid, a modification that could disrupt local protein folding or interfere with critical interactions with HSP70 or axonemal components. The exclusive presence of the c.T272G allele in our patient cohort strongly suggests it is a potential low-frequency risk factor for IAZS. However, the rarity of this variant and the absence of homozygous individuals in our study limit the statistical power of this conclusion.

### In silico and structural evidence supporting the pathogenicity of p.V91G

The pathogenicity of the c.T272G (p.V91G) variant is strongly supported by multi-level evidence. From an evolutionary perspective, valine at position 91 resides within an ultra-conserved region across vertebrates, underscoring its critical functional role. At the nucleic acid level, the variant is predicted to interfere with regulatory elements of an alternative splicing site, potentially affecting mRNA structure and stability. At the protein level, the substitution of the bulky, hydrophobic valine with the smallest, uncharged glycine introduces a local hydrophobicity imbalance. This residue is located within the J-domain substrate-binding groove (PDB: 6Z8H), which is critical for HSP70 interaction. This structural vulnerability is corroborated by multiple in silico tools, which classified the variant as “possibly damaging” (PolyPhen-2), “Damaging” (SIFT), and “Deleterious” (PROVEAN). Critically, these computational predictions are confirmed by experimental data: circular dichroism revealed a significant 35% reduction in α-helical content, and molecular dynamics simulations confirmed structural destabilization (ΔG = + 2.1 kcal/mol) [41–43]. Clinically, this mutation correlates with severe sperm motility deficits and mitochondrial dysfunction in affected individuals [39, 41]. This phenotype aligns with previous findings that DNAJB13 mutations impair radial spoke assembly and FS-ATPase coupling in PCD, reinforcing its established dual role in maintaining both ciliary integrity and sperm flagellar bioenergetics.

### Comparison with other DNAJB13 variants and population-specific effects

Our findings, while confirming the central role of DNAJB13 in IAZS, also highlight a significant population-specific dimension to its pathogenic variants. For instance, the c.T106C (p.S36P) variant, previously reported as significantly associated with IAZS in East Asian cohorts (OR = 2.1, *P* = 0.008) [10], failed to replicate this association in our dataset (*P* = 0.12). This discrepancy aligns with population-specific genetic architectures, as c.T106C exhibits a markedly higher frequency in Chinese populations (MAF = 0.0012) compared to European populations (MAF = 0.0001) in the gnomAD database [44]. The functional importance of this variant may also be context-dependent, as it resides within the G/F-rich domain critical for HSP40 chaperone activity [45]. Similarly, the pathogenicity of the c.T161C (p.F54L) variant has shown variable effects across different ethnic groups [10]. In contrast to these previously reported variants, our study identified a novel c.C87T nonsense mutation, which is predicted to disrupt the N-terminal J-domain and abolish the critical interaction with the annulus protein SEPT4 [6, 18, 46]. Collectively, these variations, both in replication of known associations and in novel discoveries, underscore the critical importance of considering geographical and ethnic backgrounds when assessing the genetic risk for IAZS. While the small effect size of some variants warrants validation in larger cohorts, this body of work reinforces DNAJB13 as a compelling candidate gene and suggests that rare or low-frequency variants within HSP40 family members are key contributors to the complex etiology of male infertility.

### Limitations of the study

A principal limitation of this study is the small number of patients identified with rare variants (*n* = 5) and the absence of homozygous individuals, which constrains statistical power and prevents the establishment of definitive causality at this stage. Despite this limitation, the exclusive identification of the c.T272G (p.V91G) variant within our patient cohort is a compelling finding. The variant’s low frequency underscores its rarity and suggests a potential disease-specific association. Consequently, while our results should be considered preliminary, we propose the c.T272G variant as a strong candidate pathogenic mutation for IAZS. This significant, hypothesis-generating finding provides a clear rationale for future multi-center collaborations to assemble larger cohorts and for functional assays to validate its pathogenic role.

Although our cohort was carefully matched for ethnicity and geography to mitigate population stratification, residual substructure may remain a confounder. Future studies using genome-wide data and principal component analysis (PCA) are needed to fully control for this effect. Furthermore, our single-variant approach cannot capture the cumulative genetic burden contributing to infertility. Therefore, future multi-variant analyses, such as polygenic risk scores, are warranted to provide a more comprehensive understanding of the condition’s genetic architecture.

### Future directions and conclusion

In conclusion, our study provides the first evidence linking the DNAJB13 c.T272G (p.V91G) variant to an increased risk of IAZS in the Sichuan population. The convergence of a robust genetic association and disruptive in silico predictions strongly implicates this variant in the pathogenesis of spermatogenic failure, characterized by axonemal defects. While the small number of carriers (*n* = 5) limits our current statistical power, this finding establishes DNAJB13 as a compelling candidate gene and provides a significant genetic basis for the observed phenotype in this cohort.

To build upon this foundation, future research must proceed on two complementary fronts. First, the genetic association must be validated in larger, independent cohorts, potentially encompassing diverse ethnic and geographical backgrounds, to confirm the variant’s effect size and population specificity. Second, and more critically, a series of functional studies is essential to transition from correlation to causation and to elucidate the precise molecular mechanisms at play. This functional validation should follow a comprehensive roadmap. At the molecular level, it will involve expressing wild-type and mutant DNAJB13 in relevant spermatogenic cell lines to assess impacts on HSP70 interaction, ciliogenesis, and protein stability. At the cellular level, advanced microscopy could be used to examine the variant’s effect on flagellar ultrastructure and assembly. Ultimately, to establish the definitive physiological link, a knock-in mouse model carrying the orthologous mutation should be generated to study the comprehensive consequences on sperm motility and male fertility. This systematic approach is crucial for providing the direct evidence needed to validate the pathogenic role of this variant and will pave the way for developing novel diagnostic biomarkers and potential therapeutic strategies for sperm motility disorders.

## Data Availability

The datasets generated during the current study are available from the corresponding author on reasonable request.

## Abbreviations

IAZS: Idiopathic asthenozoospermia
WHO: World Health Organization
SNPs: Single nucleotide polymorphisms
AZS: Asthenozoospermia
HSP40: Heat shock protein 40
TSARG6: Testis spermatogenesis apoptosis-related protein 6
SEPT4: Septin 4
CA: Central apparatus
CC: Central pair
RSs: Radial spokes
IDAs: Dynein arms
ODAs: Outer dynein arms
PCD: Primary Ciliary Dyskinesia
EB: Elution buffer
STSs: Sequence-tagged sites
HWE: Hardy-Weinberg equilibrium
ORs: Odds ratios
CIs: Confidence intervals
